# Bilateral Keratomalacia From Vitamin A Deficiency in Pancreatic Insufficiency

**DOI:** 10.7759/cureus.27569

**Published:** 2022-08-01

**Authors:** Sara Bijan, Oliver R Filutowski, Sara Safari

**Affiliations:** 1 Ophthalmology, University of South Florida, Tampa, USA; 2 Medicine, University of South Florida (USF) Health Morsani College of Medicine, Tampa, USA

**Keywords:** pancreatic insufficiency, vitamin a, xerophthalmia, keratomalacia, corneal ulcer, cornea

## Abstract

While vitamin A deficiency is a leading cause of blindness globally, it is uncommon in the developed world. Here we describe the unique presentation of a young man in the United States with keratomalacia from vitamin A deficiency related to pancreatic insufficiency. The patient presented with bilateral blurry vision that persisted for two weeks, significant unintentional weight loss, orthostatic hypotension, and profuse diarrhea. Upon slit-lamp examination, bilateral corneal opacities were appreciated. After completing additional testing, it became clear that the patient's corneal opacities were related to vitamin A deficiency from pancreatic insufficiency.

## Introduction

Vitamin A is a fat-soluble vitamin absorbed through the intestine as carotene or retinol and stored in the liver. At any given moment, the liver has a two-year supply of vitamin A. Vitamin A plays an important role in ocular surface epithelial cell differentiation and mucin production as well as the transduction of light to electrochemical energy [1,2]. Although uncommon in the developed world, vitamin A deficiency can be seen in cases of poor diet, absorption, or metabolism. Insufficient intake is often seen in patients with alcoholism, eating disorders, and other psychiatric disorders [1], while malabsorptive causes include Crohn’s disease, intestinal surgery, and pancreatic dysfunction including cystic fibrosis [2].

The ocular manifestations of vitamin A deficiency are termed xerophthalmia and include conjunctival and corneal xerosis, Bitot spots, keratomalacia, and nyctalopia [1,2]. Nyctalopia is usually the first ophthalmic symptom of xerophthalmia [2]. In more advanced disease, corneal infection and perforation can occur [3]. Current treatment options for vitamin A deficiency include dietary supplements, intramuscular administration of Vitamin A, and treating the underlying cause of the deficiency [4]. Here we describe a unique presentation of a young man with keratomalacia from vitamin A deficiency related to pancreatic insufficiency.

## Case presentation

**Figure 1:**
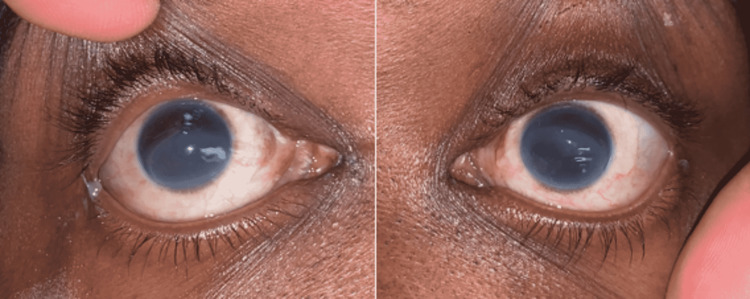
Bilateral corneal opacifications with sparing of the superior corneas in the distribution of the upper eyelids.

A 32-year-old African American male with a history of a prior abdominal gunshot wound that did not require a gastrointestinal tract repair presented to the emergency room with two weeks of bilateral vision loss. Prior to presentation, he had been experiencing months of unintentional weight loss (60lbs) and orthostatic hypotension in the setting of profuse diarrhea. At that time, he did not report night vision difficulties. His visual acuity was count fingers in both eyes and intraocular pressures via Tonopen were 9 mmHg in the right eye and 5 mmHg in the left eye. Slit lamp examination revealed left greater than right corneal stromal opacities involving the inferior 60-80% of the corneas with epithelial defects in the same distribution and associated stromal edema in both eyes (Figure 1). Mild corneal thinning was noted in the left eye. The conjunctiva were quiet, yet xerotic with no Bitot spots in either eye. Direct visualization of the posterior segment was obscured by the opacifications. B-scan ultrasound was unremarkable.

In this patient with profound weight loss and symmetric corneal opacities and epithelial defects in quiet and xerotic eyes, vitamin A deficiency was suspected from his significant malnourishment. Corneal bacterial cultures, gram stain, and herpes simplex virus (HSV) swabs were obtained. Topical moxifloxacin drops six times daily and erythromycin ointment three times daily was initiated in both eyes. Serum vitamin A levels were obtained and empiric vitamin A and zinc supplementation were advised. Corneal microbial testing returned negative. Vitamin A, C, D, and zinc levels were all low with a vitamin A level of 22 mcg/dL (normal >28 mcg/dL).

**Figure 2:**
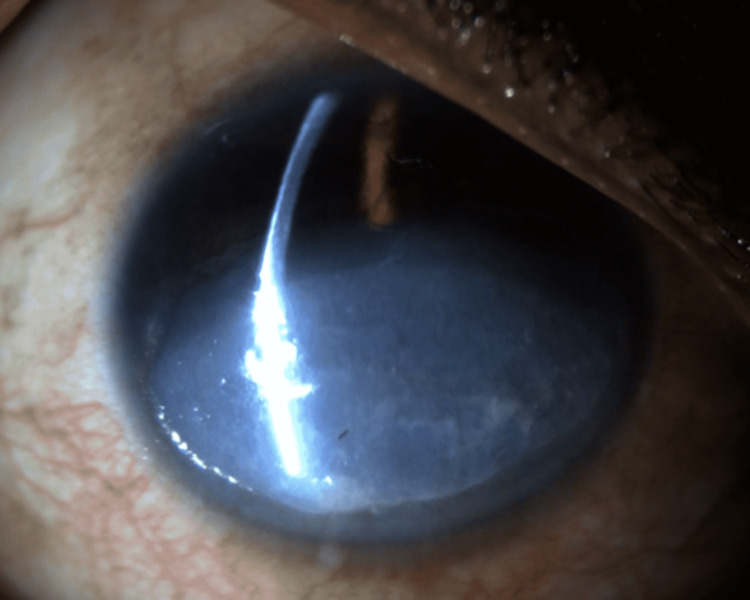
Slit lamp photograph of the right eye on the third day of hospitalization demonstrating progression in the density of the corneal opacification with new stromal thinning.

**Figure 3:**
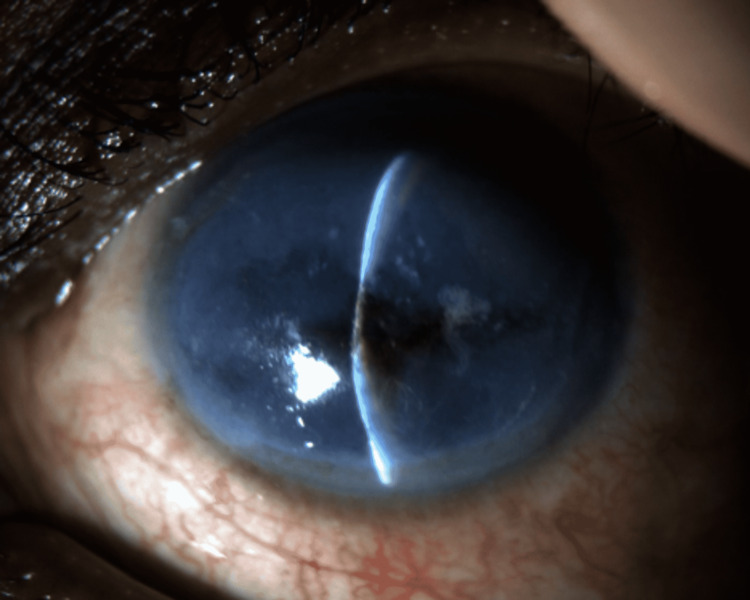
Slit lamp photograph of the left eye on the third day of hospitalization demonstrating central thinning with pigment staining of the cornea and a flat anterior chamber.

**Figure 4:**
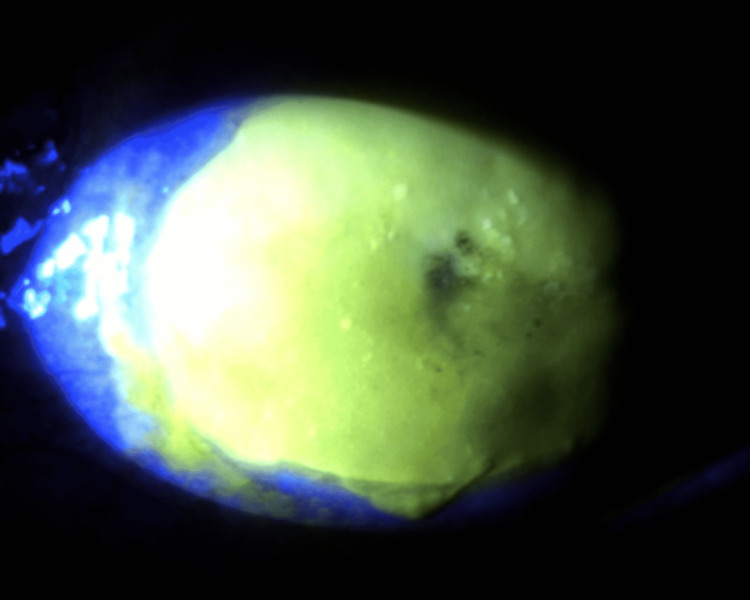
Fluorescein staining of the left eye on the third day of hospitalization. A complete epithelial defect of the cornea with a central microperforation can be observed.

Three days later, the patient's visual acuity was stable in the right eye and light perception in the left eye. Slit lamp examination revealed severe thinning with a new central microperforation and flat anterior chamber in the left eye and progression of the corneal opacification with new thinning in the right eye (Figures 2, 3, 4). Pachymetry was unavailable. The microperforation was glued and a Kontur lens (Kontur Kontact Lens Co., Inc., Hercules, California, United States) was placed on the left eye. A bandage contact was also placed on the right eye. The previously prescribed moxifloxacin drops and erythromycin ointment were stopped. Tobramycin drops four times daily and cyclopentolate drops twice daily were initiated in both eyes in addition to oral doxycycline 100 mg twice daily and oral vitamin C 1 g twice daily. Oral vitamin A supplementation (25,000 U) was continued while the patient remained hospitalized. Intramuscular and intravenous vitamin A were unavailable as they were not on the formulary at our institution.

The patient underwent an upper and lower endoscopy in the workup for his diarrhea, which revealed *Helicobacter pylori* gastritis and candida esophagitis. Stool studies were positive for *Plesiomonas shigelloides*. HIV testing was negative. He was treated with a host of antibiotics and antifungals with modest improvement. Additional labs revealed pancreatic insufficiency with a low fecal elastase. Although uncommon in African Americans, the patient had a brother with cystic fibrosis. A sweat chloride test was attempted but he was unable to perspire sufficiently to complete the test. Over the course of the hospitalization, the patient’s vision modestly improved. Once his microperforation closed, epithelial defects and corneal opacities improved, and normal vitamin A levels were achieved, the patient was discharged with outpatient follow-up.

A few days after discharge, the patient returned to the hospital after a syncopal episode related to orthostatic hypotension from dehydration. His anterior segment examination was unchanged. The patient's vision in the right eye was 20/160 and 20/500 in the left eye, which had improved since his first admission. A second sweat chloride test was performed and yielded intermediate results. Genetic testing for cystic fibrosis was negative. After a short hospitalization, he was discharged home where, a few days later, he suffered a cardiac arrest and passed away.

## Discussion

Here we provide a unique presentation of a patient with pancreatic insufficiency leading to vitamin A deficiency and keratomalacia. Vitamin A deficiency in developing countries is commonly due to limited access to vitamin A-rich food. However, in the developed world, inadequate intake is less often due to inadequate access and more often due to alcoholism, eating disorders, or psychiatric illness impairing consumption [5-8]. Fat-soluble vitamin malabsorptive disorders impairing fat-soluble vitamin retention also cause vitamin A deficiency. Our patient’s deficiency was secondary to pancreatic insufficiency. Pancreatic enzyme replacement, improved nutrition, and caloric supplements are methods to help treat vitamin A deficiency in patients with pancreatic insufficiency, especially when related to cystic fibrosis [8-10].

Vitamin A is a fat-soluble vitamin and retinoid [10]. Vitamin A plays a critical role in ocular health and visual functioning. It is a necessary component of rhodopsin, a light-sensitive protein found in the retina involved in visual phototransduction [9-12]. Vitamin A influences non-squamous corneal and conjunctival epithelial differentiation and proliferation and goblet cell functioning. Deficiency in vitamin A leads to inadequate aqueous tear and goblet cell mucin production and impaired keratinization of the epithelium. This promotes xerosis (dryness) of the ocular surface that can lead to punctate epithelial erosions and, in rare circumstances, perforation [5-7].

Xerophthalmia can also manifest as keratomalacia: corneal softening due to liquefactive necrosis (Greek κέρατο, kérato = horn; μαλακός, malakos = soft) [6]. Keratomalacia is seen in severe vitamin A deficiency and is associated with a 50% mortality rate [5,6]. Our patient presented with corneal opacifications characteristic of keratomalacia, which rapidly perforated in a few days. According to WHO guidelines, individuals with xeropthalmia should be given large oral doses of 200,000 IU vitamin A [13]. In the context of malabsorption, oral vitamin A supplementation is usually ineffective and intramuscular vitamin A supplementation is recommended with a focus on addressing the underlying malabsorptive process [5,6]. While intramuscular vitamin A increased our patient’s vitamin A levels and improved the corneal opacities and epithelial defects, the few days needed to return his vitamin A levels to normal provided enough time for rapid progression and perforation. Topical antibiotics are recommended for corneal lesions to prevent infection [13,14]. Surgical treatments for corneal perforation include glue, pedicle conjunctival flap, amniotic membrane, grafts, and transplants [15]. Additionally, doxycycline is recommended to inhibit collagenase and vitamin C supplementation is recommended for improved collagen synthesis, particularly in those who are vitamin C deficient [16-18].

## Conclusions

In conclusion, even in the developed world, vitamin A deficiency should be considered in patients with bilateral corneal opacities and suspected in those with any reason to have inadequate intake or insufficient absorption. Restoring vitamin A to normal levels is critical; however, the underlying malabsorptive processes must be addressed to ensure long-term ocular surface stability.
